# Selective stimulation of nociceptive small fibers during intraepidermal electrical stimulation: Experiment and computational analysis

**DOI:** 10.3389/fnins.2022.1045942

**Published:** 2023-01-13

**Authors:** Yuki Niimi, Jose Gomez-Tames, Toshiaki Wasaka, Akimasa Hirata

**Affiliations:** ^1^Department of Electrical and Mechanical Engineering, Nagoya Institute of Technology, Nagoya, Japan; ^2^Center for Frontier Medical Engineering, Chiba University, Chiba, Japan; ^3^Center of Biomedical Physics and Information Technology, Nagoya Institute of Technology, Nagoya, Japan

**Keywords:** intraepidermal electrical stimulation, Aδ-fiber, C-fiber, selective stimulation, multiscale electrical model, synaptic effect

## Abstract

Electrical stimulation of skin nociceptors is gaining attention in pain research and peripheral neuropathy diagnosis. However, the optimal parameters for selective stimulation are still difficult to determine because they require simultaneous characterization of the electrical response of small fibers (Aδ- and C-fibers). In this study, we measured the *in vivo* electrical threshold responses of small fibers to train-pulse stimulation in humans for the first time. We also examined selective stimulation *via* a computational model, which combines electrical analysis, and terminal fiber and synaptic models, including the first cutaneous pain C-fiber model. Selective stimulation of small fibers is performed by injecting train-pulse stimulation *via* coaxial electrodes with an intraepidermal needle tip at varying pulse counts and frequencies. The activation Aδ- or C-fibers was discriminated from the differences in reaction time. Aδ-fiber elicited a pinpricking sensation with a mean reaction time of 0.522 s, and C-fiber elicited a tingling sensation or slight burning itch with a mean reaction time of 1.243 s. The implemented multiscale electrical model investigates synaptic effects while considering stimulation waveform characteristics. Experimental results showed that perception thresholds decreased with the number of consecutive pulses and frequency up to convergence (five pulses or 70 Hz) during the selective stimulation of Aδ- and C-fibers. Considering the synaptic properties, the optimal stimulus conditions for selective stimulation of Aδ- vs. C-fibers were train of at least four pulses and a frequency of 40–70 Hz at a pulse width of 1 ms. The experimental results were modeled with high fidelity by incorporating temporal synaptic effects into the computational model. Numerical analysis revealed terminal axon thickness to be the most important biophysical factor affecting threshold variability. The computational model can be used to estimate perception thresholds while understanding the mechanisms underlying the selective stimulation of small fibers. The parameters derived here are important in exploring selective stimulation between Aδ- and C-fibers for diagnosing neuropathies.

## 1. Introduction

Human skin contains sensory receptors such as large myelinated Aβ-fibers, thin myelinated Aδ-fibers, and ultrathin unmyelinated C-fibers, each of which recognizes a different sensation (Roudaut et al., 2012; Abraira and Ginty, 2013; Djouhri, 2016). Aβ-fibers endings are low-threshold mechanoreceptors in the dermal layer and are involved in non-noxious mechanosensation, such as touch and pressure. In contrast, Aδ- and C-fibers endings are high-threshold mechanoreceptors in the epidermal layer and are involved in mechanical pain sensation.

Therefore, in recent years, selective stimulation of small fibers with terminals in the same epidermis (Aδ- and C-fibers) has been used in basic studies on nociception, neuropathic pain, and neuropathy diagnosis (Koga et al., 2005; Kodaira et al., 2014; Omori et al., 2017). Several approaches for selective stimulation have been developed, including the use of electrical, mechanical, and chemical agents (Baumgärtner et al., 2012).

One promising method for using electrical agents is intraepidermal electrical stimulation (IES), in which a localized weak current is applied through a needle electrode piercing the skin surface (Inui and Kakigi, 2012). IES is useful in pain research and diagnosis because it is easy to manage and use, noninvasive, and can predominantly stimulate painful nerves on the skin surface. Recently, pain thresholds and pain-related evoked potentials have been measured using IES in patients with diabetic neuropathy (Kukidome et al., 2016; Omori et al., 2017). In IES, different stimulation parameters were explored in experimental studies toward the selective stimulation of Aδ- and C-fibers, including duration, rising time, inter-stimulus interval of stimulation current, and electrode polarity (Inui and Kakigi, 2012; Kodaira et al., 2014; Hugosdottir et al., 2017; Omori et al., 2017).

Identification of activated fibers has been based on differences in perceived sensation (Lefaucheur et al., 2021), or on measurements of the reaction times or evoked potential latencies considering the dependency of conduction velocity on fiber thickness (Kodaira et al., 2014; Hugosdottir et al., 2017). However, it is difficult to choose the parameters for selective stimulation of Aδ- and C-fibers systematically considering a large number of factors, such as stimulation site, biophysical electrical properties of the skin, temporal/spatial neural activation integrated into the synaptic processes, and individual differences affecting pain responses (Inui et al., 2002, 2006; Otsuru et al., 2009; Patrick et al., 2018).

To overcome the above-mentioned difficulty, the first approach was to perform electrical analysis in a microscopic skin (volume conductor) model to evaluate the induced electric field intensity in IES (Mørch et al., 2011; Frahm et al., 2013; Motogi et al., 2016). A multiscale model, which integrates the computational model of the biophysical nerve model with electrical analysis, can be used to estimate the stimulation threshold for a single-pulse stimulation with different durations in Aδ-fiber activation (Hennings et al., 2017; Poulsen et al., 2020; Hirata et al., 2021; Tanaka et al., 2021b). To estimate measured perception thresholds with multiple pulse stimulation (Lilly et al., 1952; Gomez-Tames et al., 2019), we proposed a novel multiscale model integrated with the synaptic effect (Tanaka et al., 2021a). The model describes how the perception thresholds of Aδ-fiber vary for different numbers of pulses. Only a few nerve models have been reported for C-fibers in nonhuman subjects (Neishabouri et al., 2014) and humans (Tigerholm et al., 2014), and none for cutaneous pain modeling, limiting the development of selective stimulation for Aδ- and C-fibers.

In this study, to characterize synaptic effects, we measured the perception thresholds of Aδ- and C-fibers for different train pulses by considering from the differences in perceived sensation and reaction time. In addition, we propose a multiscale synaptic model that describes the responses of both Aδ- and C-fibers *via* a comparison with experiments that can be used for the first time to achieve selective activation of small fibers.

## 2. Model and methods

### 2.1. Experimental design

Two experiments were conducted to measure and compare the responses of Aδ- and C-fibers to train-pulse stimulation for the first time. One experiment evaluated the effect of the number of pulses on the perception threshold, and the other experiment investigated the variation caused by frequency (the interval of pulse current). Eight healthy participants were recruited for each experiment, six of whom participated in both experiments.

In the first experiment (eight healthy participants, age 22.0 ± 0.5 years old, six males and two females), a square wave with a pulse duration of 1 ms was used as the input current. The number of pulses varied (1, 2, 3, 4, 5, 7, and 10) at a frequency of 30 Hz.

In the second experiment (eight healthy participants, age 22.3 ± 0.7 years old, seven males and one female), the frequency varied (20, 30, 40, 50, 70, 100, and 150 Hz) for four square pulses. The order of the stimulation conditions was chosen randomly during the experiments. The Ethical Committee of the Nagoya Institute of Technology approved the experiments (No. 29–014).

### 2.2. Experimental setup

**FIGURE 1:**
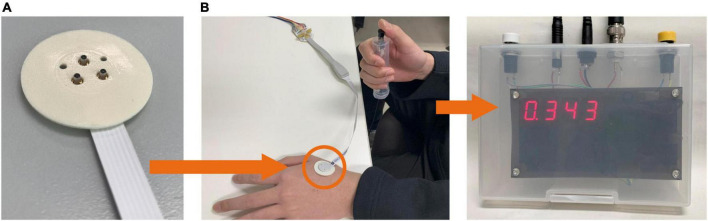
Experimental setup. **(A)** The stimulation device delivers an electric current *via* a concentric bipolar needle electrode attached to the left hand. **(B)** The participant presses a button during the perception threshold experiment to record the reaction time.

Perception thresholds for Aδ- and C-fibers were investigated for different stimulation current parameters. Figure 1 shows the experimental setup. The stimulation device (STG4004, Multi-Channel Systems GmbH, Germany) delivered multiple square pulse currents through a concentric bipolar needle electrode (NM-983 W, Nihon Kohden, Tokyo, Japan). The inner needle and ring electrodes were assigned to the anode and cathode of the stimulation device, respectively. The stimulation current was applied to the dorsum of the left hand to stimulate Aδ- or C-fibers.

### 2.3. Experimental protocol

**FIGURE 2:**
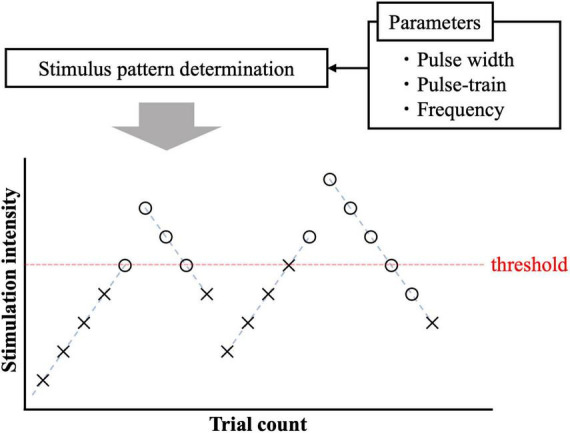
Pain threshold detection using ascending and descending trials. The circle and cross signs represent cases with and without response.

The experimental protocol to measure the perception threshold (the lowest current to generate sensation in participants) was based on the limit methods in both experiments (Figure 2). Four trials (ascending and descending) were performed per participant. In the ascending trials, the injection current intensity increased in steps of 0.02 mA up to the response. If a response occurred in the ascending trials, the current intensity was increased by 0.05 mA, and the trial was changed to descending. The injection current intensity of the descending trials was decreased in steps of 0.02 mA up to detection. If a response occurred in the descending trials, the current intensity was decreased by 0.05 mA, and the experiment continued with ascending. The participants were instructed to press a button immediately after they felt the sensation in each stimulation. To reduce uncertainty and obtain a more reliable perceptual threshold, the response was detected only if it could be felt at least twice out of three times. Then, the mean of the perceptual thresholds of the four trials was obtained as the stimulus threshold. The timing between the three stimuli was performed without informing the subjects, and the resting time between sets of three stimuli was 1 min. The maximum stimulation electric current was fixed at 1 mA. There are two reasons for upper limit of the injection current: (i) to prevent the risk of activating larger nerve fibers; (ii) because the subjects could not tolerate the pain. The time from stimulus onset to perceiving and pressing the button (termed reaction time) was automatically recorded. Each nerve has a different conduction velocity due to the presence or absence of myelination and different fiber diameters (Abraira and Ginty, 2013). The C-fibers with the smallest fiber diameter have a conduction velocity of 0.2∼2 m/s, followed by the Aδ-fibers with the largest fiber diameter of 5∼30 m/s, and the Aβ-fibers with the largest fiber diameter of 16∼100 m/s. Therefore, we used reaction time to identify the firing fiber. Based on experimental pain-related evoked potentials and reaction times after IES (Ragé et al., 2010; Kodaira et al., 2014), reaction times between 200 and 800 ms were considered for Aδ-fibers, and those exceeding 800 ms were considered for C-fibers. At the same time, we also asked the subjects to report the quality of sensation they felt.

### 2.4. Multiscale modeling of small fibers

A computational multiscale model of small fibers incorporates synaptic effects generated by train-pulse stimulation.

#### 2.4.1. Skin volume conductor modeling

**FIGURE 3:**
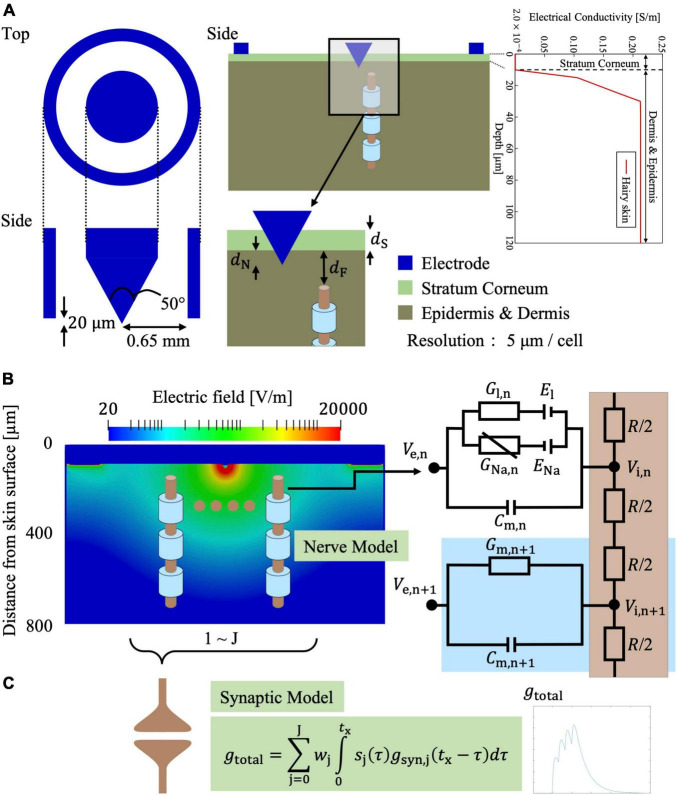
Multiscale electrical stimulation model with synaptic effects for intraepidermal electrical stimulation (IES) of small fibers (Aδ-fiber in the illustration). **(A)** IES electrode and layered skin model. **(B)** Electric field distribution in the skin on a transversal plane for an injection current of 20 μA. **(C)** The afferent spikes of the stimulated small fiber are computed and integrated into a synaptic model.

Figure 3A shows that the skin model is modeled as a layered passive volume conductor (Alekseev and Ziskin, 2007; Schmid et al., 2013; De Santis et al., 2015; Motogi et al., 2016). It is composed of three layers: stratum corneum, epidermis, and dermis. In the hairy skin, the thickness of the stratum corneum layer was 5–20 μm. The epidermis thickness was reported as 50–70 μm (Valentin, 2002; Koster et al., 2012). The conductivity values and dimensions [1.54 mm (depth) × 1.65 mm × 1.65 mm] of the skin were the same as those reported in our previous studies (Tanaka et al., 2021b). In detail, the conductivity of the stratum corneum was selected as 2 × 10^–4^ [S/m] at 2.1 kHz based on (Gabriel et al., 1996). In the transition region between the epidermis and dermis (10–30 μm), the variation of the conductivity was approximately proportional to the water content (Faes et al., 1999; Egawa and Kajikawa, 2009). The conductivity of the dermis at sufficient depth was 0.21 S/m (Wake et al., 2016). The inner electrode and ring electrode of IES corresponded to the cathode and anode, respectively, and were modeled as perfect conductors.

The scalar potential φ was solved numerically using the scalar potential finite difference method, considering that the frequency is below the kHz range and the displacement current is negligible (Trevor and Stuchly, 1996; Hirata et al., 2013):


(1) ∇⁡(σ⁢∇⁡(φ))=0

where σ is the tissue conductivity. The potential was solved iteratively using successive-over-relaxation and multigrid methods (Laakso and Hirata, 2012). A current source was connected between the inner and outer rings of the bipolar needle electrode (Motogi et al., 2014). To obtain the electric fields, the potential difference between the nodes of the voxel was divided by the voxel length.

#### 2.4.2. Nerve activation modeling for small fibers

Neural stimulation was investigated based on compartmental nerve fibers using the general Equation 2 (McNeal, 1976; Rattay, 1999) that is driven by the extracellular potential (*V*_*e*_ = φ) from Equation 1.


(2) $$\begin{array}{l} C_{n}\frac{dV_{m,n}}{dt}=-I_{ion,n}+\frac{V_{m,n-1}-2V_{m,n}+V_{m,n+1}}{0.5(R_{n}+R_{n+1})}\\ \;\;\;\;\;\;\;+\frac{V_{e,n-1}-2V_{e,n}+V_{e,n+1}}{0.5(R_{n}+R_{n+1})} \end{array}$$

The parameter *C*_*n*_ is the membrane capacitance, and *R*_*n*_ and *R*_*n*+_*_1_* are membrane resistivity of neighboring compartments. The membrane potential *V*_*m,n*_, is the difference between the extracellular potential (*V*_*e*_) and intracellular potential (*V*_*i*_).

In this study, a C-fiber model was implemented for cutaneous pain to consider thresholds for train-pulse stimulation for the first time. The C-fiber was modeled with only Ranvier nodes, which have ionic channels as nonlinear conductance with no myelin sheath present. The ionic membrane current was formulated using a modified Chiu–Ritchie–Rogart–Stagg–Sweeney model, which is a conductance-based voltage-gated model (Sweeney et al., 1987). Axon diameters (unmyelinated nerves) with an average of 1.08 ± 0.15 μm have been reported (Reilly et al., 1997). The axon diameter for the C-fiber models was chosen as 1.08 μm. Due to the difficulty in performing strength–duration experiments using single-pulse stimuli, the axonal portion was assumed to have the same parameters as the axonal part of Aδ-fibers, considering their free endings in the epidermis.

For the Aδ-fiber model, myelinated fiber compartments are internodes (myelin segments) and Ranvier nodes (ionic channels) (Figure 3B). The model is based on the developed Aδ-fiber model from our previous studies that fitted the experimental strength–duration experiment for single-pulse stimulation (Tanaka et al., 2021a,b).

Table 1 lists the electrical parameters of the Aδ- and C-fibers. The Aδ-fiber diameter was 1.69 μm (axon and myelination), considering that the ratio of axon diameter to myelin sheath diameter was approximately 0.6 (Gillespie and Stein, 1983). The depths of the Aδ- and C-fiber terminals were 25 and 10 μm, respectively, from the stratum corneum bottom. Since nerve endings are distributed perpendicularly to the skin surface (MacIver and Tanelian, 1993; Hilliges et al., 1995), these nerve models were placed in the same way.

**TABLE 1:** Electrical parameters of myelinated and non-myelinated nerves.

Parameter	Value	
	**Aδ-fiber**	**C-fiber**
Nernst potential for sodium channels (*E*_*Na*_)	115 mV	
Nernst potential for leakage channels (*E*_*l*_)	–0.01 mV	
Sodium channel conductance (*G*_*Na*_)	1445 mS/cm^2^	
Leaked channel conductance (*G*_*l*_)	17.92 mS/cm^2^	
Capacitance of membrane (myelinated) (*C*_*n*_)	1.00 pF	–
Membrane resistance (myelinated) (*R*_*n*_)	129 MΩ	–
Myelin conductance (*G*_*m*_)	0.045 nS	–
Capacitance of membrane (non-myelinated) (*C*_*n+1*_)	1.12 pF	
Membrane resistance (non-myelinated) (*R*_*n+1*_)	1.15 MΩ	

#### 2.4.3. Synaptic model

In our previous study (Tanaka et al., 2021a), we proposed a new synaptic model. The model is briefly summarized. The synergistic effects of train-pulse electrical stimulation were investigated in a synaptic model (Figure 3C). The action potentials from Aδ- or C-fibers produce an excitatory postsynaptic current that can change the transmembrane potential of the postsynaptic neuron. If the transmembrane membrane potential reaches a minimum threshold, an action potential is fired in the postsynaptic neuron. To begin, synaptic conductance is modeled as the difference of two exponentials that is a general function adopted to describe the observed experimental synaptic conductance profiles (Roth and van Rossum, 2013):


(3) $$g_{j}=g_{m\,a\,x,j}\,f\,\left(e^{-\frac{t}{\tau_{f,j}}}-e^{-\frac{t}{\tau_{r,j}}}\right)$$

where *g*_*maxx,j*_ is the peak conductance, and τ_*r,j*,_ and τ_*f,j*_ are the rise and fall time constants, respectively. The normalization factor *f* (Roth and van Rossum, 2013) is set such that the amplitude equals *g*_*maxx,j*_.

A total synaptic conductance *g*_*total*_ in the postsynaptic neuron is calculated by combining the effects of each synapse *j* at time *t*_*x*_ and the spike sequences (action potentials) *s*_*j*_ arriving from the presynaptic neuron (Aδ- and C-fibers), as follows:


(4) $$g_{t\,o\,t\,a\,l}\,\left(t_{x}\right)=\sum_{j=0}^{J}\omega_{j}\,\int_{0}^{t_{x}}s_{j}\,\left(\tau \right)\,g_{j}\,\left(t_{x}-\tau \right)\,d\tau$$

where *J* is the total number of presynaptic neurons (Aδ- and C-fibers), and *s*_*j*_ is the delta pulse. The probability of depolarizing the synapse is considered by the weighting term *w*_*j*_. Then, the excitatory postsynaptic transmembrane current (EPSC) is given as follows:


(5) $$E\,P\,S\,C\,\left(t_{x}\right)=g_{t\,o\,t\,a\,l}\,\left(t_{x}\right)\,\left[E-V_{m}\,\left(t_{x}\right)\right]$$

where *E* is the postsynaptic reversal potential and *Vm* is the postsynaptic membrane potential. This represents the current that passes through a synaptic channel that induces depolarization in the postsynaptic cell.

We evaluated the synaptic responses in a postsynaptic neuron using an Izhikevich spike model (Izhikevich, 2003).

The parameters in the synaptic effect model (Table 2) were fitted by comparison results considering the spatial information (the number of activated presynaptic neurons) and temporal information (spiking sequence) to activate postsynaptic neurons under different train-pulse conditions for C- and Aδ-fibers.

**TABLE 2:** Estimated synaptic model parameters based on the measured results.

Parameter	Value	
	**Aδ-fiber**	**C-fiber**
Rise time constant (τ_r_)	6 ms	6 ms
Fall time constant (τ_f_)	11 ms	58 ms
Synaptic weight (ω)	6.1 × 10^3^	7.5 × 10^3^

(1) Activation of multiple fiber terminals is required to elicit a conscious perception (Mouraux et al., 2012). The relationship between the stimulation threshold and the number of stimulated fibers is obtained using the multiscale model, considering that more fibers are activated at higher injection currents, assuming a uniform fiber density. Therefore, the number of presynaptic neurons was based on the reported fiber densities of Aδ-fiber (160 fibers/mm^2^) and C-fiber (640 fibers/mm^2^) (Ebenezer et al., 2007) multiplied by the stimulation region for each current threshold. The maximum number of fibers connected to the synapse were 212 and 849 fibers, considering the maximum area of the needle ring, for Aδ- and C-fiber, respectively.
(2) The injection current required to activate the postsynaptic neuron was computed for different numbers of train pulses and frequencies. The parameters space comprising the synaptic weights (ω), rise time (τ_r_), and fall time (τ_f_) of the synaptic conductance were adjusted to minimize the least-squares error between the experimental and computational injection current threshold, as proposed in Gomez-Tames et al. (2019) (Table 2).

## 3. Results

### 3.1. Experimental threshold dependence on pulse number and frequency

The train-pulse stimulation was investigated for different number of pulses and frequencies. The measured perception threshold values (and therefore reaction times) corresponded to C-fiber or Aδ-fiber. In the ascending trial, the subjects first perceived a delayed sensation like a tingle or itch (C-fiber) and then an earlier sensation like a pinprick (Aδ-fiber). In the descending trial, the subjects first perceived an earlier sensation like a pinprick (Aδ-fiber) then a delayed sensation like a tingle or itch (C-fiber) and then the subjects felt nothing. Table 3 summarizes the reaction times of the subjects in each condition. Overall, the mean reaction time for the Aδ-fiber was 0.522±0.122 s and that of the C-fiber was 1.243 ± 0.290 s. Reaction time was varied in average from 0.522 to 1.243 s or vice versa with increasing or decreasing stimulus intensity.

**TABLE 3:** Reaction time of the perception threshold at different numbers of pulses (frequency of 30 Hz) and frequencies (pulse number of four) in Aδ- and C-fibers.

Pulse number	Reaction time [s] (Mean ± SD)		Frequency [Hz]	Reaction time [s] (Mean ± SD)	
	**Aδ-fiber**	**C-fiber**		**Aδ-fiber**	**C-fiber**
1	0.546 ± 0.163	–	20	0.460 ± 0.104	1.060 ± 0.149
2	0.504 ± 0.136	1.274 ± 0.372	30	0.489 ± 0.070	1.160 ± 0.220
3	0.554 ± 0.131	1.276 ± 0.365	40	0.475 ± 0.095	1.170 ± 0.266
4	0.578 ± 0.154	1.327 ± 0.326	50	0.473 ± 0.116	1.136 ± 0.138
5	0.542 ± 0.134	1.443 ± 0.344	70	0.487 ± 0.088	1.256 ± 0.243
7	0.550 ± 0.109	1.305 ± 0.342	100	0.512 ± 0.106	1.336 ± 0.345
10	0.595 ± 0.130	1.255 ± 0.316	150	0.542 ± 0.139	1.167 ± 0.202

**FIGURE 4:**
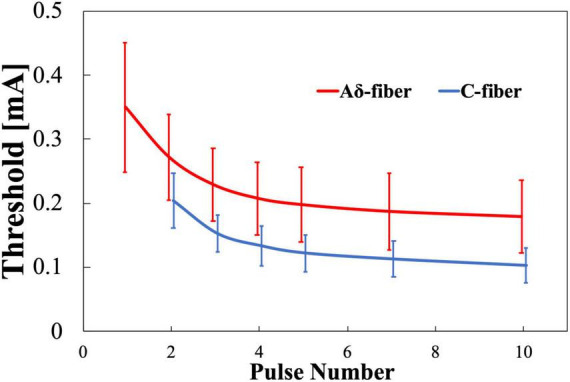
Experimental pain threshold (mean value and standard deviation, *n* = 8). Variation with pulse number in Aδ- and C-fibers. Note that there was no detection of C-fiber activation at single-pulse stimulation.

The experimental thresholds dependence on pulse number variation of Aδ- and C-fibers were obtained for train-pulse stimulation with a different number of pulses at a fixed frequency of 30 Hz. Figure 4 shows that the thresholds of both nerves decreased and converged to a minimum value with increasing the number of stimulation pulses. The difference in the threshold between 7 and 10 stimulation pulses was smaller than 10%. For single-pulse stimulation, Aδ-fiber response was observed in all subjects, but not for C-fiber, in which six of the eight participants were not confirmed. The thresholds of the Aδ-fiber exceeded those of the C-fiber when the stimulation pulse number was two or more. The threshold normalized by a minimum threshold (average between the 7 and 10 pulses of stimulation) was 1.6 and 1.9 times higher at the two pulses than the minimum threshold for Aδ- and C-fibers, respectively (Supplementary Figure 1). The Aδ-fiber threshold for single-pulse stimulation was two times higher than the minimum threshold. The difference in the threshold between Aδ- and C-fibers was notable for pulse numbers exceeding three.

**FIGURE 5:**
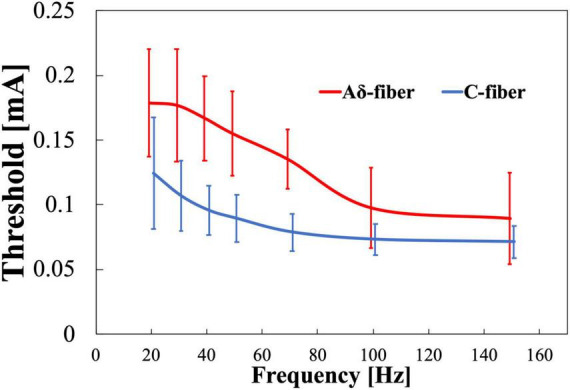
Experimental pain threshold (mean value and standard deviation, *n* = 8). Variation with frequency in Aδ- and C-fibers.

The experimental thresholds dependence on frequency of Aδ- and C-fibers were obtained for train-pulse stimulation at a different frequency with a fixed number of stimulation pulses (four pulses). Figure 5 shows that the thresholds of both nerves decreased and converged to a minimum value with increasing frequency. Thresholds were consistently higher for the Aδ-fiber. The difference in the converged thresholds was 10% between 100 and 150 Hz for both fibers. The threshold normalized by a minimum threshold (average between 100 and 150 Hz) was 2.1 and 1.7 times higher at 20 Hz than the minimum threshold for Aδ- and C-fibers, respectively (Supplementary Figure 2). The difference in the threshold between Aδ- and C-fibers was notable at 30–70 Hz.

For the stimulation conditions common to both experiments (four pulses and 30 Hz), the thresholds of Aδ-fiber and C-fiber in the first experiment (Figure 4) were 0.208 ± 0.057 and 0.133 ± 0.032 mA, respectively, and the thresholds for Aδ-fiber and C-fiber in the second experiment (Figure 5) were 0.177 ± 0.043 and 0.107 ± 0.027 mA, respectively. In overall, the results of the second experiment were lower overall than the results of the first one, in part because the subjects were different in both experiments.

### 3.2. Multiscale electrical stimulation model with the synaptic effect of small fibers

**FIGURE 6:**
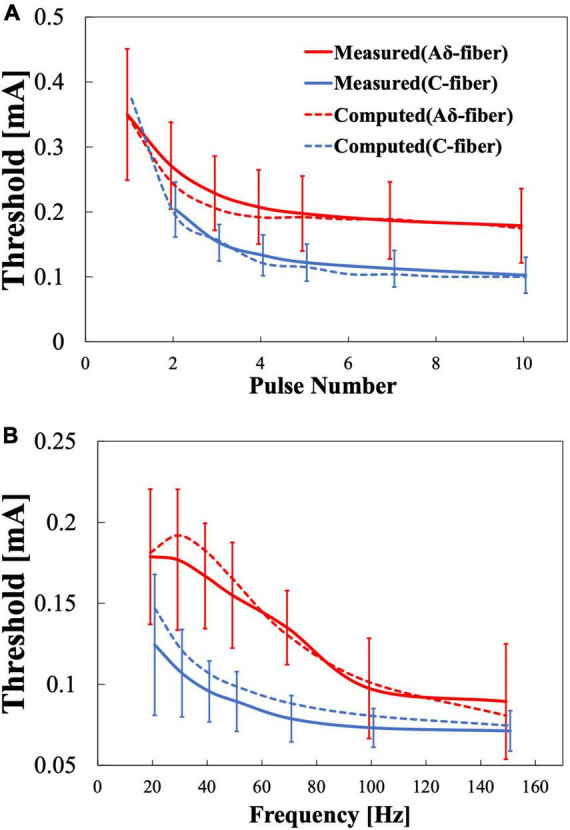
Multiscale electrical stimulation model results for pain thresholds. **(A)** Variation with the number of stimulation pulses when the frequency is fixed at 30 Hz. **(B)** Variation with frequency when the stimulation pulse number is fixed at 4.

Figure 6 shows the computational results for the perception threshold using a set of parameters regarding the synaptic effect. The root mean squared error (RMSE) of the experimental and computational results was 12 and 11 μA for the Aδ- and C-fibers, respectively.

### 3.3. Factors related to selective stimulation and threshold variability

Factors affecting stimulation thresholds related to experimental conditions and anatomical differences were examined computationally. Table 4 lists the parameter variations and the corresponding maximum variations of the computed injection current thresholds (MacIver and Tanelian, 1993; Reilly et al., 1997; De Santis et al., 2015). Variations in the depth needle electrode, stratum corneum thickness, and depth of the fiber terminal modified the threshold up to 3% for both fibers, whereas the variation in the fiber diameter altered the threshold by 22–37%. Variation with fiber diameter was higher in Aδ-fibers than C-fibers, similar to the observed higher variability of experimental stimulation thresholds in Aδ-fibers (Figure 4).

**TABLE 4:** Maximum variation in the computed injection current thresholds for the reported range values of the simulation parameters.

Parameter	Range [μm]	Value [μA]	
		**Aδ-fiber**	**C-fiber**
Needle depth (*d_N_*)	5, 10, 20*, 30	1.08 (0.3%)	3.79 (2.7%)
Stratum corneum thickness (*d_S_*) (Motogi et al., 2016)	5, 10*, 15, 20	3.82 (1.6%)	2.06 (0.6%)
Nerve terminal depth (*d_F_*) (MacIver and Tanelian, 1993)	10, 25*, 40 (Aδ)	4.80 (2.1%)	0.85 (0.01%)
	5, 10*, 15 (C)		
Fiber diameter (Reilly et al., 1997)	1.45, 1.69*, 1.92 (Aδ)	129 (37%)	97 (26%)
	0.93, 1.08*, 1.23 (C)		

*Values used in Section “3.3. Factors related to selective stimulation and threshold variability” for fitting experimental results. Values within parentheses are the maximum variation normalized by the experimental threshold value.

## 4. Discussion

This is the first study to investigate the perception thresholds of Aδ- and C-fibers using IES with varying numbers of consecutive pulses and frequencies to consider temporal and spatial summation factors in the synaptic process affecting pain perception (Koga et al., 2005; Roth and van Rossum, 2013). A multiscale model with a synaptic effect was also implemented. It features the first unmyelinated fiber for cutaneous pain that describes the experimental responses of the C-fiber. Furthermore, the Aδ-fiber and synaptic models developed in a previous study corroborated the new experimental results presented here (Tanaka et al., 2020, 2021a). The model allows for a deeper evaluation of the mechanism and an examination of the optimal stimulation.

In our experiment, differences in perceived sensation and conduction velocity between Aδ- and C-fibers were used to distinguish the stimulated fiber (Weiss et al., 2008; Lefaucheur et al., 2021). The measured response times corroborated other IES studies of the hand (0.51 ± 0.10 [s] for Aδ-fibers and 1.46 ± 0.38 [s] for C-fibers) (Omori et al., 2017; Table 3). The reduction of the reaction time associated with successive increases in stimulus intensity indicates that C-fiber were activated firstly (lower threshold) and then Aδ-fiber is added at higher stimulation intensities. When the response of Aδ- is added, the reaction time will be given by the faster reaction of Aδ-fiber. However, we cannot discard or confirm (and to a what degree) if the delayed nociceptive information from C-fiber (when C- and Aδ- are activated) is integrated in some way into the pain perception.

In the first experiment (Figure 4), the thresholds of both Aδ- and C-fibers decreased to a convergent value as the number of pulses increased. This is due to the synaptic effects of putative postsynaptic neurons in the anterior horn of the spinal cord, as shown in previous studies (Tanaka et al., 2021a). Experimental results for Aδ-fibers (Supplementary Figure 1) showed that single-pulse stimulation was 2.0 ± 0.5 times the convergence value at a pulse width of 1 ms and a fixed frequency (30 Hz). This tendency was found to be similar to a previous study (Tanaka et al., 2021a) that used a pulse width of 400 μs and a fixed frequency of 30 Hz. The C-fiber presented a higher variation in the stimulation thresholds within the number of pulses investigated, which may reflect stronger synaptic effects.

In the second experiment (Figure 5), thresholds of both Aδ- and C-fibers decreased to convergent values as the frequency increased in agreement with a higher temporal summation of postsynaptic potentials, as the neurotransmitters released from synaptic vesicles cause depolarization more frequently and a temporary increase in the membrane potential. The electrical stimulation conditions for this series of pulses in our study are similar to those that elicit flexion reflexes (Sandrini et al., 2005). However, since the intensity was at the sensory threshold and the subject did not show any response to retract his hand, it was assumed that no reflex was produced.

The multiscale model includes these synaptic effects in good agreement with the experimental results. The computational model was fitted simultaneously using the results of the two experiments (stimulus pulse number variation and frequency variation). For a common stimulation condition in both experiments (four pulses and 30 Hz), there was a 16 and 22% percentage difference between Aδ- and C-fibers, respectively. The RMSE could be reduced if the parameters were fitted for each experiment (12–9 μA and 11–5 μA for Aδ- and C-fiber, respectively).

C-fiber activation for single-pulse stimulation could not be detected in six out of eight participants (Otsuru et al., 2009; Motogi et al., 2014). The lack of detection of C-fibers for single-pulse stimulation could be explained if Aδ-fibers have a smaller threshold at single-pulse stimulation. In this case, the reaction time would correspond only to faster Aδ-fibers, even if C-fiber is activated with enough injection currents. This was confirmed by the computational model, which showed a higher stimulation threshold prediction for C-fiber in single-pulse stimulation conditions (Figure 6A). The computational results showed that the threshold for single-pulse stimulation of the C-fiber (373 μA) exceeded the Aδ-fiber threshold (349 μA).

We also revised the stimulation-related factors that may explain why C-fiber could be detected in two out of eight subjects. In particular, we investigated the effect of needle electrode depth and anatomical differences (tissue thickness, nerve diameter, and fiber terminal position) (Motogi et al., 2016). Needle depth and stratum corneum thickness affect the potential distribution by IES, nerve terminal depth affects the retrieved external potential when calculated by the nerve model, and fiber diameter affects the electrical parameters of the nerve. The dominant factor affecting the stimulation threshold was solely the fiber thickness for the parameter variation considered here. Decreasing both fiber diameters increased the perceptual threshold. In this case, the Aδ-fiber threshold surpassed the C-fiber threshold, even with single-pulse stimulation. Aδ-fiber threshold did not preclude C-fiber activation in two subjects. Experimental values of two subjects who showed a C-fiber response to single-pulse stimulation were compared with numerical analysis using two fiber diameters: original (Aδ-fiber: 1.69 μm, C-fiber: 1.08 μm) and smaller (Aδ-fiber: 1.45 μm, C-fiber: 0.93 μm) (Supplementary Figures 3A, B, respectively). From the comparison of the figures, the threshold for a smaller axon diameter was closer to the experimental results for the two subjects. The RMSE of the experimental and computed values for the two subjects decreased from 49 to 9 μA for the Aδ-fiber and from 27 to 14 μA for the C-fiber using a smaller axon diameter.

Given the variability of the experimental and computed results of the multiscale model, among the conditions performed in this study, the optimal stimulus conditions for selective stimulation of Aδ- vs. C-fibers are train of at least four pulses and a frequency of 40–70 Hz at a pulse width of 1 ms. Stimulation parameters in IES mainly include electrode polarity, stimulation waveform (square wave, triangular wave, or trapezoidal wave), pulse width, pulse number, frequency, number of electrode pairs, and electrode size. Although some preliminary experiments have been conducted, the number of parameters is large and optimal conditions have not yet been proposed (Otsuru et al., 2009, 2010; Kodaira et al., 2014; Motogi et al., 2014). Since it is very difficult to search for optimal conditions experimentally, it is desirable to use a computer model to do so, and remained a challenging topic for the future.

If not selective stimulation of Aδ- and C-fibers, the optimal parameters of stimulation to reduce the threshold of nociceptive fiber activation should be train of at least four pulses and at least 100 Hz. This is similar to the stimulus parameters for eliciting flexion reflexes (RIII reflex) (Sandrini et al., 2005).

A limitation of the proposed model is that the target of the threshold estimation is the dorsum of the hand. Parameters may need to be changed if other sites are targeted for stimulation. Future study is expected to use these nerve activation and synaptic models to investigate the optimal electrode conditions for the selective stimulation of peripheral nerves and to apply them to peripheral neuropathy diagnosis.

## Data availability statement

The raw data supporting the conclusions of this article will be made available by the authors, without undue reservation.

## Ethics statement

The studies involving human participants were reviewed and approved by Ethical Committee of the Nagoya Institute of Technology (No. 29−014). The patients/participants provided their written informed consent to participate in this study.

## Author contributions

AH and TW conceived and designed the research. TW and YN conducted the experiments. JG-T and YN conducted the experiments and processed the data. All authors analyzed the data, wrote the manuscript, read, and approved the submitted version.

## References

[B1] Abraira V. E. Ginty D. D. (2013). The sensory neurons of touch. *Neuron* 79 618–639. 10.1016/j.neuron.2013.07.051 23972592 PMC3811145

[B2] Alekseev S. I. Ziskin M. C. (2007). Human skin permittivity determined by millimeter wave reflection measurements. *Bioelectromagnetics* 28 331–339. 10.1002/bem.20308 17429851

[B3] Baumgärtner U. Greffrath W. Treede R. D. (2012). Contact heat and cold, mechanical, electrical and chemical stimuli to elicit small fiber-evoked potentials: Merits and limitations for basic science and clinical use. *Neurophysiol. Clin.* 42 267–280. 10.1016/J.NEUCLI.2012.06.002 23040698

[B4] De Santis V. Chen X. L. Laakso I. Hirata A. (2015). An equivalent skin conductivity model for low-frequency magnetic field dosimetry. *Biomed. Phys. Eng. Exp.* 1:015201. 10.1088/2057-1976/1/1/015201

[B5] Djouhri L. (2016). Aδ-fiber low threshold mechanoreceptors innervating mammalian hairy skin: A review of their receptive, electrophysiological and cytochemical properties in relation to Aδ-fiber high threshold mechanoreceptors. *Neurosci. Biobehav. Rev.* 61 225–238. 10.1016/j.neubiorev.2015.12.009 26746589

[B6] Ebenezer G. J. Hauer P. Gibbons C. McArthur J. C. Polydefkis M. (2007). Assessment of epidermal nerve fibers: A new diagnostic and predictive tool for peripheral neuropathies. *J. Neuropathol. Exp. Neurol.* 66 1059–1073. 10.1097/nen.0b013e31815c8989 18090915

[B7] Egawa M. Kajikawa T. (2009). Changes in the depth profile of water in the stratum corneum treated with water. *Skin Res. Technol.* 15 242–249. 10.1111/j.1600-0846.2009.00362.x 19622134

[B8] Faes T. J. C. van der Meij H. A. de Munck J. C. Heethaar R. M. (1999). The electric resistivity of human tissues (100 HZ-10 MHZ): A meta- analysis of review studies. *Physiol. Measure.* 20:201. 10.1088/0967-3334/20/4/201 10593226

[B9] Frahm K. S. Mørch C. D. Grill W. M. Lubock N. B. Hennings K. Andersen O. K. (2013). Activation of peripheral nerve fibers by electrical stimulation in the sole of the foot. *BMC Neurosci.* 14:116. 10.1186/1471-2202-14-116 24103294 PMC3851563

[B10] Gabriel S. Lau R. W. Gabriel C. (1996). The dielectric properties of biological tissues: III. Parametric models for the dielectric spectrum of tissues. *Phys. Med. Biol.* 41 2271–2293. 10.1088/0031-9155/41/11/003 8938026

[B11] Gillespie M. J. Stein R. B. (1983). The relationship between axon diameter, myelin thickness and conduction velocity during atrophy of mammalian peripheral nerves. *Brain Res.* 259 41–56. 10.1016/0006-8993(83)91065-X 6824935

[B12] Gomez-Tames J. Hirata A. Tamura M. Muragaki Y. (2019). Corticomotoneuronal model for intraoperative neurophysiological monitoring during direct brain stimulation. *Int. J. Neural Syst.* 29 1–14. 10.1142/S0129065718500260 30037285

[B13] Hennings K. Frahm K. S. Petrini L. Andersen O. K. Arendt-Nielsen L. Mørch C. D. (2017). Membrane properties in small cutaneous nerve fibers in humans. *Muscle Nerve* 55 195–201. 10.1002/MUS.25234 27366884

[B14] Hilliges M. Wang L. Johansson O. (1995). Ultrastructural evidence for nerve fibers within all vital layers of the human epidermis. *J. Invest. Dermatol.* 104 134–137. 10.1111/1523-1747.ep12613631 7798631

[B15] Hirata A. Diao Y. Onishi T. Sasaki K. Ahn S. Colombi D. (2021). Assessment of human exposure to electromagnetic fields: review and future directions. *IEEE Trans. Electromagn. Compatibil.* 63 1619–1630. 10.1109/TEMC.2021.3109249

[B16] Hirata A. Ito F. Laakso I. (2013). Confirmation of quasi-static approximation in SAR evaluation for a wireless power transfer system. *Phys. Med. Biol.* 58 N241—-N249. 10.1088/0031-9155/58/17/N241 23939244

[B17] Hugosdottir R. Mørch C. D. Andersen O. K. Helgason T. Arendt-Nielsen L. (2017). Evaluating the ability of non-rectangular electrical pulse forms to preferentially activate nociceptive fibers by comparing perception thresholds. *Scand. J. Pain* 16 175–175. 10.1016/J.SJPAIN.2017.04.032

[B18] Inui K. Kakigi R. (2012). Pain perception in humans: Use of intraepidermal electrical stimulation. *J. Neurol. Neurosurg. Psychiatry* 83 551–556. 10.1136/jnnp-2011-301484 22138180

[B19] Inui K. Tran T. D. Hoshiyama M. Kakigi R. (2002). Preferential stimulation of Aδ fibers by intra-epidermal needle electrode in humans. *Pain* 96 247–252. 11972996 10.1016/S0304-3959(01)00453-5

[B20] Inui K. Tsuji T. Kakigi R. (2006). Temporal analysis of cortical mechanisms for pain relief by tactile stimuli in humans. *Cereb. Cortex* 16 355–365. 10.1093/CERCOR/BHI114 15901650

[B21] Izhikevich E. M. (2003). Simple model of spiking neurons. *IEEE Trans. Neural Netw.* 14 1569–1572. 10.1109/TNN.2003.820440 18244602

[B22] Kodaira M. Inui K. Kakigi R. (2014). Evaluation of nociceptive Aδ- and C-fiber dysfunction with lidocaine using intraepidermal electrical stimulation. *Clin. Neurophysiol.* 125 1870–1877. 10.1016/j.clinph.2014.01.009 24566074

[B23] Koga K. Furue H. Rashid M. H. Takaki A. Katafuchi T. Yoshimura M. (2005). Selective activation of primary afferent fibers evaluated by sine-wave electrical stimulation. *Mol. Pain* 1 1–11. 10.1186/1744-8069-1-13 15813963 PMC1083421

[B24] Koster M. I. Loomis C. A. Koss T. K. (2012). “Skin development and maintenance,” in *Dermatology*, 3rd Edn, eds Bolognia J. L. Jorizzo J. L. Schaffer J. V. (Philadelphia, PA: Elsevier Saunders), 55–64.

[B25] Kukidome D. Nishikawa T. Sato M. Igata M. Kawashima J. Shimoda S. (2016). Measurement of small fibre pain threshold values for the early detection of diabetic polyneuropathy. *Diabetic Med.* 33 62–69. 10.1111/dme.12797 25970541

[B26] Laakso I. Hirata A. (2012). Fast multigrid-based computation of the induced electric field for transcranial magnetic stimulation. *Phys. Med. Biol.* 57 7753–7765. 10.1088/0031-9155/57/23/7753 23128377

[B27] Lefaucheur J. P. Abbas S. A. Lefaucheur-Ménard L. Rouie D. Tebbal D. Bismuth J. (2021). Small nerve fiber selectivity of laser and intraepidermal electrical stimulation: A comparative study between glabrous and hairy skin. *Neurophysiol. Clin.* 51 357–374. 10.1016/j.neucli.2021.06.004 34304975

[B28] Lilly J. C. Austin G. M. Chambers W. W. (1952). Threshold movements produced by excitation of cerebral cortex and efferent fibers with some parametric regions of rectangular current pulses (cats and monkeys). *J. Neurophysiol.* 15 319–341. 14955703 10.1152/jn.1952.15.4.319

[B29] MacIver M. B. Tanelian D. L. (1993). Structural and functional specialization of Aδ and C fiber free nerve endings innervating rabbit corneal epithelium. *J. Neurosci.* 13 4511–4524. 10.1523/jneurosci.13-10-04511.1993 8410200 PMC6576377

[B30] McNeal D. R. (1976). Analysis of a model for excitation of myelinated nerve. *IEEE Trans. Biomed. Eng.* 4 329–337. 10.1109/tbme.1976.324593 1278925

[B31] Mørch C. D. Hennings K. Andersen O. K. (2011). Estimating nerve excitation thresholds to cutaneous electrical stimulation by finite element modeling combined with a stochastic branching nerve fiber model. *Med. Biol. Eng. Comput.* 49 385–395. 10.1007/s11517-010-0725-8 21207174

[B32] Motogi J. Kodaira M. Muragaki Y. Inui K. Kakigi R. (2014). Cortical responses to C-fiber stimulation by intra-epidermal electrical stimulation: an MEG study. *Neurosci. Lett.* 570 69–74. 24731795 10.1016/j.neulet.2014.04.001

[B33] Motogi J. Sugiyama Y. Laakso I. Hirata A. Inui K. Tamura M. (2016). Why intra-epidermal electrical stimulation achieves stimulation of small fibres selectively: a simulation study. *Phys. Med. Biol.* 61 4479–4490. 10.1088/0031-9155/61/12/4479 27223492

[B34] Mouraux A. Ragé M. Bragard D. Plaghki L. (2012). Estimation of intraepidermal fiber density by the detection rate of nociceptive laser stimuli in normal and pathological conditions. *Neurophysiol. Clin.* 42 281–291. 10.1016/J.NEUCLI.2012.05.004 23040699

[B35] Neishabouri A. Faisal A. A. Budd J. (2014). Saltatory conduction in unmyelinated axons: clustering of Na + channels on lipid rafts enables micro-saltatory conduction in C-fibers. *Front. Neuroanat.* 8:109. 10.3389/fnana.2014.00109 25352785 PMC4195365

[B36] Omori S. Isose S. Misawa S. Watanabe K. Sekiguchi Y. Shibuya K. (2017). Pain-related evoked potentials after intraepidermal electrical stimulation to Aδ and C fibers in patients with neuropathic pain. *Neurosci. Res.* 121 43–48. 10.1016/j.neures.2017.03.007 28322984

[B37] Otsuru N. Inui K. Yamashiro K. Miyazaki T. Ohsawa I. Takeshima Y. (2009). Selective stimulation of C fibers by an intra-epidermal needle electrode in humans. *Open Pain J.* 2 53–56. 10.2174/1876386300902010053

[B38] Otsuru N. Inui K. Yamashiro K. Miyazaki T. Takeshima Y. Kakigi R. (2010). Assessing Aδ fiber function with lidocaine using intraepidermal electrical stimulation. *J. Pain* 11 621–627. 10.1016/j.jpain.2009.10.001 20075012

[B39] Patrick E. E. Currlin S. Kundu A. Delgado F. Fahmy A. Madler R. (2018). Design and assessment of stimulation parameters for a novel peripheral nerve interface. *IEEE Eng. Med. Biol. Soc.* 2018 5491–5494. 10.1109/EMBC.2018.8513582 30441580

[B40] Poulsen A. H. Tigerholm J. Meijs S. Andersen O. K. Mørch C. D. (2020). Comparison of existing electrode designs for preferential activation of cutaneous nociceptors. *J. Neural Eng.* 17:036026. 10.1088/1741-2552/ab85b1 32235064

[B41] Ragé M. Van Acker N. Facer P. Shenoy R. Knaapen M. W. Timmers M. (2010). The time course of CO2 laser-evoked responses and of skin nerve fibre markers after topical capsaicin in human volunteers. *Clin. Neurophysiol.* 121 1256–1266. 10.1016/j.clinph.2010.02.159 20347388

[B42] Rattay F. (1999). The basic mechanism for the electrical stimulation of the nervous system. *Neuroscience* 89 335–346. 10077317 10.1016/s0306-4522(98)00330-3

[B43] Reilly D. M. Ferdinando D. Johnston C. Shaw C. Buchanan K. D. Green M. R. (1997). The epidermal nerve fibre network: Characterization of nerve fibres in human skin by confocal microscopy and assessment of racial variations. *Br. J. Dermatol.* 137 163–170. 10.1046/j.1365-2133.1997.18001893.x 9292061

[B44] Roth A. van Rossum M. C. W. (2013). “Modeling synapses,” in *Computational Modeling Methods for Neuroscientists*, Ed. De Schutter E. (Cambridge, MA: MIT Press), 139–160. 10.7551/mitpress/9780262013277.003.0007

[B45] Roudaut Y. Lonigro A. Coste B. Hao J. Delmas P. Crest M. (2012). Touch sense: functional organization and molecular determinants of mechanosensitive receptors. *Channels* 6 234–245. 10.4161/chan.22213 23146937 PMC3508902

[B46] Sandrini G. Serrao M. Rossi P. Romaniello A. Cruccu G. Willer J. C. (2005). The lower limb flexion reflex in humans. *Progr. Neurobiol.* 77 353–395. 10.1016/j.pneurobio.2005.11.003 16386347

[B47] Schmid G. Cecil S. Überbacher R. (2013). The role of skin conductivity in a low frequency exposure assessment for peripheral nerve tissue according to the ICNIRP 2010 guidelines. *Phys. Med. Biol.* 58 4703–4716. 10.1088/0031-9155/58/13/4703 23774744

[B48] Sweeney J. D. Mortimer J. T. Durand D. (1987). “Modeling of mammalian myelinated nerve for functional neuromuscular electrostimulation,” in *Proceedings of the IEEE 97th Annual Conf. Engineering in Medicine Biology Society*, Vol. 9 Boston, 1577–1578.

[B49] Tanaka S. Gomez-Tames J. Inui K. Ueno S. Hirata A. Hirata A. (2021a). Synaptic effect of Aδ-Fibers by pulse-train electrical stimulation. *Front. Neurosci.* 15:643448. 10.3389/FNINS.2021.643448/FULL PMC8107290 33981196

[B50] Tanaka S. Gomez-Tames J. Wasaka T. Inui K. Ueno S. Hirata A. (2021b). Electrical characterisation of Aδ-fibres based on human in vivo electrostimulation threshold. *Front. Neurosci.* 14:588056. 10.3389/FNINS.2020.588056 33584171 PMC7873976

[B51] Tanaka T. Isomura Y. Kobayashi K. Hanakawa T. Tanaka S. Honda M. (2020). Electrophysiological effects of transcranial direct current stimulation on neural activity in the rat motor cortex. *Front. Neurosci.* 14:495. 10.3389/FNINS.2020.00495/BIBTEX PMC7340144 32714126

[B52] Tigerholm J. Petersson M. E. Obreja O. Lampert A. Carr R. Schmelz M. (2014). Modeling activity-dependent changes of axonal spike conduction in primary afferent C-nociceptors. *J. Neurophysiol.* 111 1721–1735. 10.1152/JN.00777.2012 24371290 PMC4044369

[B53] Trevor W. D. Stuchly A. M. (1996). Analytic validation of a three-dimensional scalar-potential finite-difference code for low-frequency magnetic induction. *Appl. Comput. Electromagn. Soc. J.* 11 72–81.

[B54] Valentin J. (2002). Basic anatomical and physiological data for use in radiological protection: reference values: ICRP Publication 89. *Ann. ICRP* 32 1–277. 14506981

[B55] Wake K. Sasaki K. Watanabe S. (2016). Conductivities of epidermis, dermis, and subcutaneous tissue at intermediate frequencies. *Phys. Med. Biol.* 61 4376–4389. 10.1088/0031-9155/61/12/4376 27224275

[B56] Weiss T. Straube T. Boettcher J. Hecht H. Spohn D. Miltner W. H. (2008). Brain activation upon selective stimulation of cutaneous C- and Adelta-fibers. *NeuroImage* 41 1372–1381. 10.1016/J.NEUROIMAGE.2008.03.047 18499480

