# Supplementary material for: Selective stimulation of nociceptive small fibers during intraepidermal electrical stimulation: Experiment and computational analysis
Source: Front Neurosci. 2023 Jan 13;16:1045942. doi: 10.3389/fnins.2022.1045942 (PMC9880216; doi:10.3389/fnins.2022.1045942)
Supplement: Supplementary file 1 [file Data_Sheet_1.pdf]

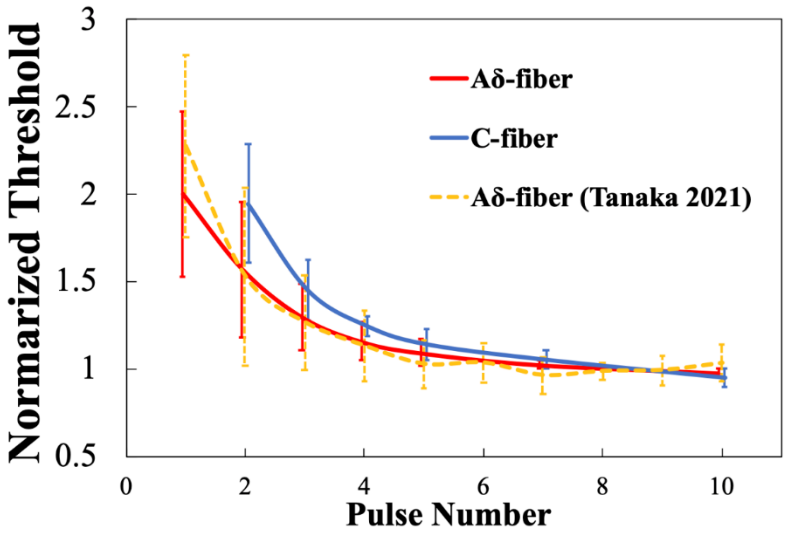

Figure S1. Normalized data by minimum threshold (average value between the seventh and tenth pulses) for the results in Figure 4.

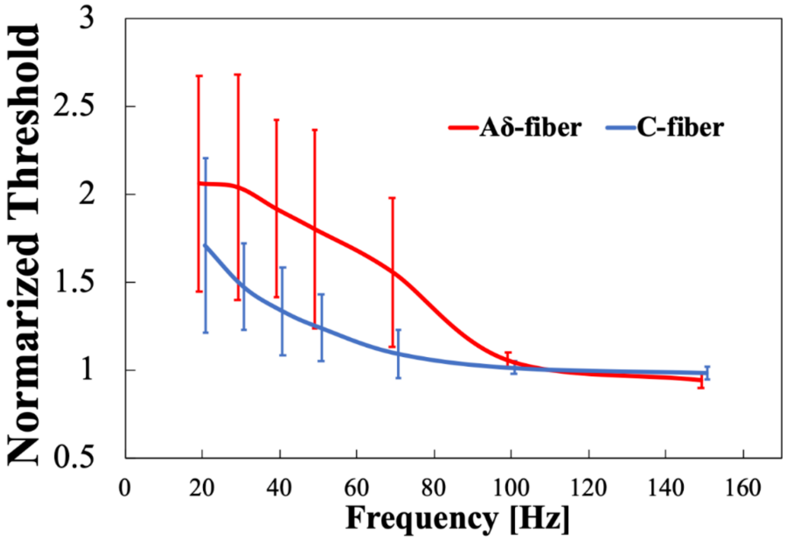

Figure S2. Normalized data by minimum threshold (average of the thresholds between 100 Hz and 150 Hz) for the results in Figure 5.

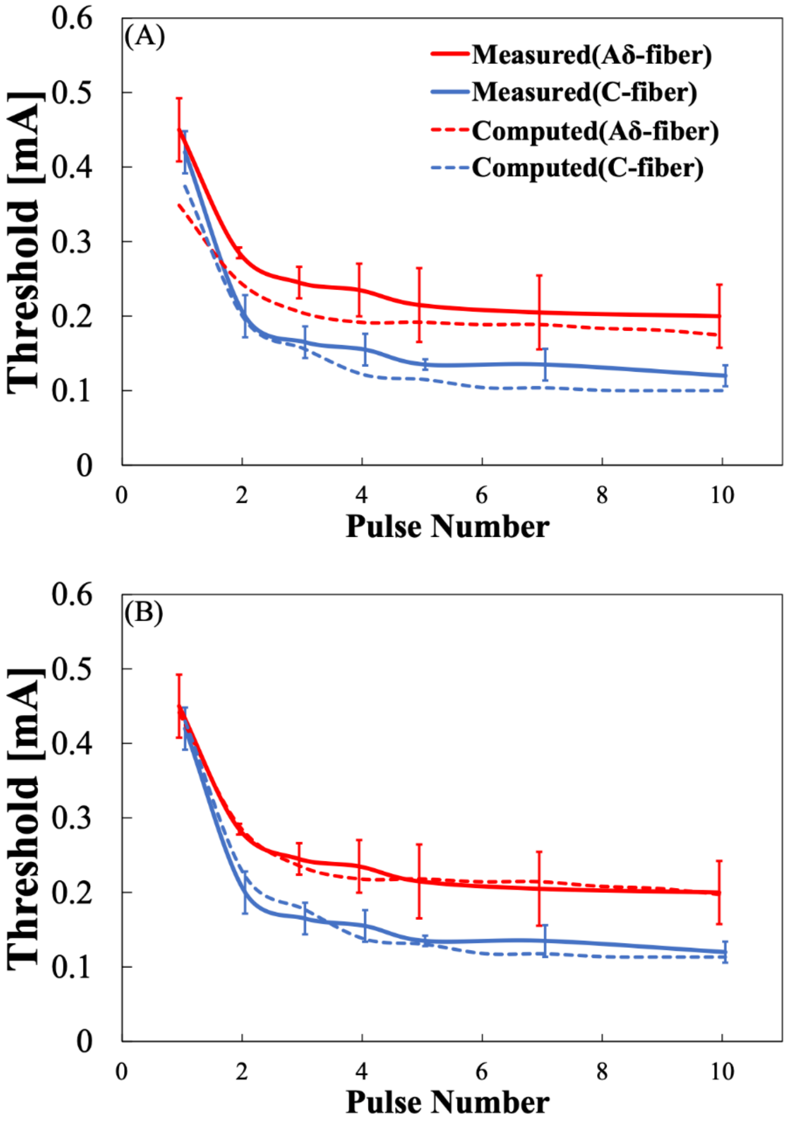

Figure S3. Experimental averaged values of two subjects with C-fiber response to single-pulse stimulation. Computed stimulation thresholds for two axon diameters: **(A)** 1.08 µm and **(B)** 0.93 µm.
